# Predicting Long-Term Stability of Precise Oscillators under Influence of Frequency Drift

**DOI:** 10.3390/s18020502

**Published:** 2018-02-07

**Authors:** Weiwei Cheng, Guigen Nie

**Affiliations:** 1 GNSS (Global Navigation Satellite System) Research Center, Wuhan University, 129 Luoyu Road, 430079 Wuhan, China; edmund1954@sina.com; 2 Collaborative Innovation Center for Geospatial Information Technology, Wuhan University, 129 Luoyu Road, 430079 Wuhan, China

**Keywords:** frequency stability, drift, power-law noise, convex optimization, compressive sensing

## Abstract

High-performance oscillators, atomic clocks for instance, are important in modern industries, finance and scientific research. In this paper, the authors study the estimation and prediction of long-term stability based on convex optimization techniques and compressive sensing. To take frequency drift into account, its influence on Allan and modified Allan variances is formulated. Meanwhile, expressions for the expectation and variance of discrete-time Hadamard variance are derived. Methods that reduce the computational complexity of these expressions are also introduced. Tests against GPS precise clock data show that the method can correctly predict one-week frequency stability from 14-day measured data.

## 1. Introduction

Timing technology is important in modern finance [1], industries and scientific research [2]. High frequency trading, real-time navigation and the verification of relativistic effects require accurate and high-resolute time and/or frequency information. Timing information is given by counting the periodic signals of referenced oscillators. Meanwhile, frequencies of the timing signal are multiples of the referenced oscillator period. A time-scale is accurate only if the participant oscillators produce frequencies consistent with their nominal values or are stable enough to be predictable. Furthermore, high resolution requires, in turn, a short period of oscillator output signal frequencies. Unfortunately, no high-performance oscillator produces constant and high-resolution signals.

The difference between an oscillator’s output signal from its nominal value can be divided into deterministic and random parts. The oscillator’s random behavior is well-documented by a class of noise processes called power-law noise (PLN) [3]. While the random variations are defined in the frequency domain, it is often measured in the time domain by a class of structure functions and referred to as the frequency stability of the oscillator. For example, Allan (AVAR), modified Allan (MVAR) and Hadamard variance (HVAR) are commonly-used methods. These statistics can be improved by using a ‘total approach’ [4]. Recently, Thêo- [5] and parabolic variances [6] were also proposed. The authors proposed an oscillator noise analysis method called stochastic ONA [7]. The method predicts long-term frequency stabilities using convex optimization techniques. Specifically, the confidence regions of long-term Hadamard variances (HVAR) predicted from 14-day GPS precise clock data include HVAR estimated from 168-day measured data and are smaller than those estimated from 84-day time derivations.

On the other hand, distinctions between deterministic and random behavior are blurry [8]. It is often difficult to differentiate drift from frequency noises [9]. A main drawback of stochastic ONA is its requirement for drift-free input variances. For example, cesium frequency standards are conventionally believed to be free from drift [10]. However, analysis of historical data and current practice show that the performance of TAI (International Atomic Time) improved when taking the frequency drifts of participant cesium clocks into account [11].

This paper studies the estimation of oscillator stability under the influence of frequency drift. In Section 2, the basic concepts and methods of time domain stability have been reviewed. Although time domain stability is related to the frequency domain, discrete sampling has different impacts on the two. The influence of discrete sampling on both domains has also been reviewed in this section. In Section 3, we introduce a method called stochastic ONA, which extends the oscillator noise analysis problem to the prediction of long-term stability. In the following section, we describe methods to compute coefficient matrices used in stochastic ONA. We also introduce a method that greatly reduces the computational complexity of Walter’s characterization of AVAR and MVAR. From these works, we can then predict long-term frequency contaminated by deterministic linear frequency drift. The proposed model is tested against GPS precise clock data in Section 5. The one-week AVAR, MVAR and HVAR predicted by stochastic ONA from 14-day measured data are consistent with those estimated from 84-day data. In addition, the fifteen-day variances predicted by stochastic ONA have more compact confidence regions than those estimated from 42–60-day data.

## 2. Review of Time Domain Stability

It is well documented that high performance oscillators are influenced by power-law noises (PLN). PLN processes are conventionally defined by their power spectral densities (PSD):

(1) $$S_{y}\left(f\right)=\sum_{i=1}^{N_{h}}h_{\alpha_{i}}f^{\alpha_{i}}=\left(2\pi f^{2}\right)S_{x}\left(f\right)$$

where $S_{y}\left(f\right)$ is the PSD of oscillator fractional frequency y(t), $S_{x}\left(f\right)$ PSD of time deviations x(t),

$$x\left(t\right)=\int_{0}^{t}y\left(t\right)dt.$$

*f* (Fourier) frequency and $h_{\alpha }$ noise intensity coefficient, $\alpha =\alpha_{1}$, $\alpha_{2},\cdots$, $\alpha_{N_{h}}$. Often, α=2 (white phase modulation, WHPM), 1 (flicker PM, FLPM), 0 (white frequency modulation, WHFM), −1 (flicker FM, FLFM), −2 (random walk FM, RWFM) [12]. However in Global Positioning System (GPS) master control station (MCS) clock prediction, α = 2, 0, −2 and −4 (random run FM, RRFM), and the $h_{\alpha }$ coefficients are replaced by $q_{i}$ [4]:

$$q_{i}=\left\{\begin{array}{cc} h_{2}/\left(8\pi^{2}\tau_{0}\right), & i=0,\\ {\left(2\pi \right)}^{2(i-1)}h_{2(1-i)}\tau_{0}, & i=1,2\;or\;3. \end{array}\right.$$

However, PSD measured from an oscillator signal is not used solely in practice. Since the PSD estimates are “noisy” [13], time domain statistics are often used instead. For instance, AVAR [13]:

(2) $${\hat{\sigma }}_{y}^{2}\left(\tau \right)=\frac{1}{2\left(N-2m\right)\tau^{2}}\sum_{i=1}^{N-2m}{\left[x_{i+2m}-2x_{i+m}+x_{i}\right]}^{2},$$

MVAR:

(3) $$\mathrm{Mod}{\hat{\sigma }}_{y}^{2}\left(\tau \right)=\sum_{j=1}^{N-3m+1}\frac{{\left[\sum_{i=j}^{j+m-1}\left(x_{i+2m}-2x_{i+m}+x_{i}\right)\right]}^{2}}{2m^{2}\tau^{2}\left(N-3m+1\right)},$$

and HVAR:

(4) $${\hat{\sigma }}_{z}^{2}\left(\tau \right)=\sum_{i=1}^{N-3m}\frac{{\left[x_{i+3m}-3x_{i+2m}+3x_{i+m}-x_{i}\right]}^{2}}{6\left(N-3m\right)\tau^{2}}.$$

A time-domain variance $\sigma_{k}^{2}\left(\tau \right)$ can be related to its PSD:

(5) $$\sigma_{k}^{2}\left(\tau \right)=\int_{0}^{\infty }S_{y}\left(f\right){\left|H_{k}\left(f\right)\right|}^{2}df=\sum_{i=1}^{N_{h}}\Phi_{k}\left(\alpha_{i},\tau \right)h_{\alpha_{i}}.$$

Here, $\tau =m\tau_{0}$ is the averaging time, $\tau_{0}$ sampling period and $H_{k}\left(f\right)$ the transfer function of $\sigma_{k}^{2}\left(\tau \right)$ defined in [12]:

(6) $$\Phi_{k}\left(\alpha ,\tau \right)=\int_{0}^{\infty }f^{\alpha }{\left|H_{k}\left(f\right)\right|}^{2}df.$$

Here, subscript *k* is used as a generic form of different variances. The majority of measured data nowadays are digital. $\sigma_{k}^{2}\left(\tau \right)$ estimated from finite data may have different values for Equation (5). The former is usually denoted as $\hat{\sigma_{k}^{2}\left(\tau \right)}$, and it can be viewed as a realization of sample variance variable $\Sigma_{k}\left(\tau \right)$ [14]. The distribution function $F\left(\Sigma_{k}\left(\tau \right)\leq {\overset{˘}{\sigma }}_{k}^{2}\left(\tau \right)\right)$ ($F\left({\overset{˘}{\sigma }}_{k}^{2}\left(\tau \right)\right)$ for short) of random variable $\Sigma_{k}\left(\tau \right)$ can be formulated as:

(7) $$F\left({\overset{˘}{\sigma }}_{k}^{2}\left(\tau \right)\right)=\int_{0}^{{\overset{˘}{\sigma }}_{k}^{2}\left(\tau \right)}\frac{u^{{\mathrm{EDF}}_{k}\left(\tau \right)/2}}{ue^{-u}\Gamma \left({\mathrm{EDF}}_{k}\left(\tau \right)/2\right)}du,$$

for arbitrary positive real number ${\overset{˘}{\sigma }}_{k}^{2}\left(\tau \right)$, where *e* is Euler’s number, Γ(·) the Gamma function, ${\mathrm{EDF}}_{k}\left(\tau \right)$ the equivalence degrees of freedom (EDF):

(8) $${\mathrm{EDF}}_{k}\left(\tau \right)=\frac{E{\left[{\hat{\sigma }}_{k}^{2}\left(\tau \right)\right]}^{2}}{\mathrm{Var}\left[{\hat{\sigma }}_{k}^{2}\left(\tau \right)\right]},$$

$E\left[\cdot \right]$ variance is estimated from infinite samples and $\mathrm{Var}\left[\cdot \right]$ variance of the random variable ${\hat{\sigma }}_{k}^{2}\left(\tau \right)$.

If we denote:

(9) $$E\left[{\hat{\sigma }}_{k}^{2}\left(\tau \right)\right]=\sum_{i=1}^{N_{h}}\Phi_{k}\left(\alpha_{i},\tau \right)h_{\alpha_{i}},$$

where variance ${\hat{\sigma }}_{k}^{2}\left(\tau \right)$ is estimated from x[t] and x[t] the discrete sampling of time deviations x(t), then $\Phi_{k}\left(\alpha_{i},\tau \right)$ does not equal Equation (6). Instead of PSD, Kasdin shows that it is the symmetric two-time autocorrelation function:

(10) $$R_{x}\left(t,\tau \right)\equiv \langle x(t-\tau /2)x(t+\tau /2)\rangle ,$$

which directly samples the continuous-time function [15]. For example, instead of Equation (1), Walter shows that PSD measured from discrete sampled time deviations x[t] relates to $S_{y}\left(f\right)$ in the following way [16]:

(11) $$S_{x}\left(f\right)=\frac{h_{\alpha }}{4\pi^{2}}{\left[\frac{\sin(\pi f\tau_{0})}{\pi \tau_{0}}\right]}^{\alpha -2}=\frac{\tau_{0}^{2}S_{y}^{d}\left(f\right)}{4\sin^{2}\left(\pi \tau_{0}f\right)}.\;$$

The autocorrelation function of PLN processes has the following asymptotic form when t>>τ [15]:

(12) $$R\left(t,\tau \right)\approx \frac{h_{\alpha }}{2{\left(2\pi \right)}^{\alpha }}\left(\log4t-\log\left|\tau \right|\right)$$

for α=−1, and:

(13) $$R_{x}\left(t,\tau \right)\approx \left\{\frac{{Q\Gamma \left(\alpha -1\right)|\tau |}^{1-\alpha }}{\Gamma (\alpha /2)\Gamma (1-\alpha /2)}+\frac{Q\Gamma \left(1-\alpha \right)t^{1-\alpha }}{\Gamma \left(2-\alpha \right)\Gamma^{2}\left(1-\alpha /2\right)}\right\}$$

for α≠−1, where:

$$Q=\frac{h_{\alpha }}{2{\left(2\pi \right)}^{\alpha }}.$$

To derive an expression for the discrete autocorrelation function, the deviation of Brownian motion is often replaced by the discrete Wiener process in time and frequency metrology [15,17]. If, in addition, the noise process is wide sense stationary, $R_{x}\left(t,\tau \right)$ can be recast as [15]:

(14) $$R_{x}^{\left(d\right)}\left[m\right]=\frac{Q\Gamma (m+1-\alpha /2)\Gamma (\alpha -1)}{\tau_{0}^{\alpha -1}\Gamma \left(m-1+\alpha /2\right)\Gamma \left(\alpha /2\right)\Gamma \left(1-\alpha /2\right)}.$$

Here, $\tau =m\tau_{0}$. From Equations (12)–(14), Walter derives $\Phi_{k}\left(\alpha_{i},\tau \right)$:

(15) $$\begin{array}{c} \begin{array}{cc} \Phi_{\mathrm{AVAR}}\left(\alpha_{i},\tau \right)=\frac{\pi \Gamma (\alpha_{i}-1)}{m^{2}{\left(2\pi \tau_{0}\right)}^{\alpha_{i}+1}\Gamma^{2}\left(\alpha_{i}/2\right)}\times & \left[3-4\frac{\Gamma \left(m+1-\alpha_{i}/2\right)\Gamma \left(\alpha_{i}/2\right)}{\Gamma \left(m+\alpha_{i}/2\right)\Gamma \left(1-\alpha_{i}/2\right)}\right.\\ & +\left.\frac{\Gamma \left(2m+1-\alpha_{i}/2\right)\Gamma \left(\alpha_{i}/2\right)}{\Gamma \left(2m+\alpha_{i}/2\right)\Gamma \left(1-\alpha_{i}/2\right)}\right] \end{array} \end{array}$$

and $\mathrm{Var}\left[{\hat{\sigma }}_{k}^{2}\left(\tau \right)\right]$:

(16) $$\begin{array}{cc} \mathrm{Var}\left[{\hat{\sigma }}_{y}^{2}\left(\tau \right)\right]= & \frac{h_{\alpha }^{2}\Gamma^{2}\left(\alpha -1\right)\sin^{2}\left(\alpha \pi /2\right)}{{\left(2\pi \tau_{0}\right)}^{2\alpha +2}\left(N-2m\right)m^{4}}\times \sum_{\ell =-N+2m+1}^{N-2m-1}\left(2-\frac{2|\ell |}{N-2m}\right)\\ & \times \left\{\frac{3\Gamma (|\ell |+1-\alpha /2)}{\Gamma (|\ell |+\alpha /2)}-\frac{2\Gamma (|m+\ell |+1-\alpha /2)}{\Gamma (|m+\ell |+\alpha /2)}\right.-\frac{2\Gamma (|m-\ell |+1-\alpha /2)}{\Gamma (|m-\ell |+\alpha /2)}\\ & +\frac{\Gamma (|2m+\ell |+1-\alpha /2)}{2\Gamma (|2m+\ell |+\alpha /2)}{\left.+\frac{\Gamma (|2m-\ell |+1-\alpha /2)}{2\Gamma (|2m-\ell |+\alpha /2)}\right\}}^{2} \end{array}$$

for Allan variance (AVAR) [16]. It should be noted that variances estimated from discrete sampled data may be distorted when the averaging time $\tau =m\tau_{0}$ is near sampling period $\tau_{0}$. The distortions are caused by alias and measurement noise [15]. Equations (15) and (16) do not take the distortions into account. Furthermore, the influences of frequency drift are not included in these equations. As a development of Walter’s work, we will formulate the effect of deterministic linear frequency drift in AVAR estimates and derive $\Phi_{k}\left(\alpha_{i},\tau \right)$ and $\mathrm{Var}\left[{\hat{\sigma }}_{k}^{2}\left(\tau \right)\right]$ for HVAR in Section 4.

On the other hand, if ${\hat{\sigma }}_{k}^{2}\left(\tau \right)$ is a measurement of the time-domain variance $\sigma_{k}^{2}\left(\tau \right)$, we can estimate the $h_{\alpha }$ coefficients from ${\hat{\sigma }}_{k}^{2}\left(\tau \right)$. It can be formed as a least square problem:

(17) $$\mathrm{minimize}\;{\left(\Phi h-\sigma \right)}^{T}W\left(\Phi h-\sigma \right),$$

and called oscillator noise analysis. Suppose there are *M* different input variances. Therefore, the coefficient matrix Φ can be divided into *M* blocks:

$$\Phi ={\left[\begin{array}{ccccc} \Phi_{k_{1}}^{T} & \cdots & \Phi_{k}^{T} & \cdots & \Phi_{k_{M}}^{T} \end{array}\right]}^{T},$$

whose *k*-th block $\Phi_{k}$, $k=k_{1}$, $k_{2},\cdots$, $k_{M}$,

(18) $$\Phi_{k}=\left[\begin{array}{ccc} \Phi_{k}\left(\alpha_{1},\tau_{0}\right) & \cdots & \Phi_{k}\left(\alpha_{N_{h}},\tau_{0}\right)\\ \Phi_{k}\left(\alpha_{1},2\tau_{0}\right) & \cdots & \Phi_{k}\left(\alpha_{N_{h}},2\tau_{0}\right)\\ \vdots & \ddots & \vdots \\ \Phi_{k}\left(\alpha_{1},m_{k}\tau_{0}\right) & \cdots & \Phi_{k}\left(\alpha_{N_{h}},m_{k}\tau_{0}\right) \end{array}\right],$$

$\Phi_{k}\left(\alpha ,\tau \right)$ is defined in Equation (9). Φ can be formed by using the method we proposed in Section 4. Similarly, the column vector of input variances σ can be partitioned into *M* blocks:

$$\sigma ={\left[\begin{array}{ccccc} \sigma_{k_{1}}^{T} & \cdots & \sigma_{k}^{T} & \cdots & \sigma_{k_{M}}^{T} \end{array}\right]}^{T},$$

the *k*-th block:

$$\sigma_{k}={\left[\begin{array}{ccccc} {\hat{\sigma }}_{k}^{2}\left(\tau_{0}\right) & \cdots & {\hat{\sigma }}_{k}^{2}\left(m\tau_{0}\right) & \cdots & {\hat{\sigma }}_{k}^{2}\left(m_{k}\tau_{0}\right) \end{array}\right]}^{T},$$

is comprised of ${\hat{\sigma }}_{k}^{2}\left(m_{k}\tau_{0}\right)$, m=1, ⋯, $m_{k}$ estimated from time deviations x[i], i=1, 2,⋯, *N*. *h* is a column vector of noise intensity coefficients and *W* the weight matrix. The works in [18,19] give different ways to compute *W*.

## 3. Stochastic ONA

We extended the oscillator noise analysis problem to the prediction of long-term stability. Since the likelihood (or conditional probability) of:

$$\sigma_{k}^{2}\left(\tau \right)=\text{specified positive real number}$$

is zero, we estimate a 1−2ε confidence region of $\sigma_{k}^{2}\left(\tau \right)$ instead. This extension is realized by using convex optimization techniques (Appendix A.2), and we call it stochastic ONA [7].

The basic idea of stochastic ONA is:

$$F\left(\sigma_{k}^{2}\left(\tau \right)<\sum_{i=1}^{N_{h}}B_{k}\left(\alpha_{i},\tau ,1-\varepsilon \right)h_{\alpha_{i}}\right)\to 1$$

and:

$$F\left(\sigma_{k}^{2}\left(\tau \right)<\sum_{i=1}^{N_{h}}B_{k}\left(\alpha_{i},\tau ,\varepsilon \right)h_{\alpha_{i}}\right)\to 0$$

when ε→0, where F(·) is the chi-square distribution function defined in Equation (7), ε>0,

(19) $$B_{k}\left(\alpha ,\tau ,\varepsilon \right)=\frac{F^{-1}\left(\varepsilon \right)\times \Phi_{k}\left(\alpha ,m\tau_{0}\right)}{{\left.{\mathrm{EDF}}_{k}\left(\tau \right)\right|}_{\alpha }}.$$

Clearly,

B(ε)h≤σ≤B(1−ε)h

when ε is small enough. The matrices B(ε) and B(1−ε) are obtained by substituting $\Phi_{k}\left(\alpha ,\tau \right)$ in the coefficient matrix Φ with $B_{k}\left(\alpha ,\tau ,\varepsilon \right)$ and $B_{k}\left(\alpha ,\tau ,1-\varepsilon \right)$. This can be cast into the following optimization problem:

(20) $$\mathrm{minimize}\;{\left(\Phi h-\sigma \right)}^{T}W\left(\Phi h-\sigma \right),$$

where the $N_{h}$-dimensional column vector of the noise intensity coefficients *h* subject to:

(21) $$\left[\begin{array}{c} B(\varepsilon )h-\sigma \\ -B(1-\varepsilon )h+\sigma \\ -h \end{array}\right]\leq 0.$$

In practice, it is not always easy to find such an ε. In addition, Equation (21) does not model the uncertainty of input variances completely. For example, neither the correlations among the different averaging time, nor those among different variances calculated from the same underlying time series are taken into account. We therein prescribe a lower bound $\varepsilon_{l}$ as a threshold. Equation (21) will be replaced by an alternative model if stochastic ONA fails to find an $\varepsilon \geq \varepsilon_{l}$ that holds for the inequalities. While different variances estimated from the same time series are correlated, they contain independent pieces of information. It is difficult to formulate the correlations of structure functions precisely. It is even more difficult to solve stochastic programming under complex probabilistic constraints. When unformulated information only has a strong impact on a small population of the whole input variances, they can be treat as ‘violations’ using the techniques of compressive sensing. The auxiliary variables μ and ν are used as indicators for the violations. Specifically, the $\sum_{i=1}^{M}m_{i}$-dimensional non-negative vector variable μ and ν are defined such that:

$$\begin{array}{c} B(\varepsilon )h\leq \mathrm{diag}(1+\mu )\sigma ,\\ B(1-\varepsilon )h\geq \mathrm{diag}(1-\nu )\sigma . \end{array}$$

Since $B\left(\varepsilon \right)h\leq E\left[\sigma \right]\leq B\left(1-\varepsilon \right)h$,

$$E\left[∥\mu ∥+∥\nu ∥\right]=0\leq \min\left\{∥\mu ∥+∥\nu ∥\right\}$$

for any norm $∥\cdot ∥$ of μ and ν. We choose the $\ell_{1}$-norm (see Appendix A.1 for details):

(22) $${∥\nu ∥}_{1}\equiv \sum_{i=1}^{\sum_{j=1}^{M}m_{j}}\left|\nu_{i}\right|$$

where $\nu_{i}$ is the *i*-th component of ν, and $|\nu_{i}|$ returns the absolute value of $\nu_{i}$. The probabilistic fact that only a minority of input variances violate Equation (21) can be formulated using the property of $\ell_{1}$: the minimum of the $\ell_{1}$ norm is approximately sparse when we have more variables than problem data. Therein, the optimization problem can be formulated as:

(23) $${\mathrm{minimize}\;∥\mu ∥}_{1}+{∥\nu ∥}_{1},$$

where optimization variables (h,μ,ν) subject to:

$$\left[\begin{array}{c} B(\varepsilon )h-\mathrm{diag}\{\sigma \}\mu -\sigma \\ -B(1-\varepsilon )h-\mathrm{diag}\{\sigma \}\nu +\sigma \\ -\mu \\ -\nu \\ \nu -1\\ -h \end{array}\right]\leq 0.$$

We adjust the values of input variance according to the result of Equation (23). Consequently, we can find that *h* holds for Equation (21) with the adjusted variances. Suppose $\left(h^{*},\mu^{*},\nu^{*}\right)$ is the optimum of Equation (23). We label an input variance ${\hat{\sigma }}_{k}^{2}\left(\tau \right)$ as an ‘outlier’ when:

- case I:
  $$\sum_{i=1}^{N_{h}}B_{k}\left(\alpha_{i},\tau ,\varepsilon \right)h_{\alpha_{i}}^{*}>{\hat{\sigma }}_{k}^{2}\left(\tau \right);$$
- case II:
  $$\sum_{i=1}^{N_{h}}B_{k}\left(\alpha_{i},\tau ,1-\varepsilon \right)h_{\alpha_{i}}^{*}<{\hat{\sigma }}_{k}^{2}\left(\tau \right).$$

An outlier will be adjusted in the following way:

(24) $$\begin{array}{c} \left\{\begin{array}{c} \sum_{i=1}^{N_{h}}\left[\left(1-\psi \right)B_{k}\left(\alpha_{i},\tau ,\varepsilon \right)+\psi \Phi_{k}\left(\alpha_{i},\tau \right)\right]h_{\alpha_{i}}^{*},\;\text{case I};\\ \sum_{i=1}^{N_{h}}\left[\left(1-\psi \right)B_{k}\left(\alpha_{i},\tau ,1-\varepsilon \right)+\psi \Phi_{k}\left(\alpha_{i},\tau \right)\right]h_{\alpha_{i}}^{*},\;\text{case II}, \end{array}\right. \end{array}$$

where 0<ψ<1. We set ψ = 0.5 in this article.

By then, we can either minimize or maximize the values of:

(25) $$\sum_{i=1}^{N_{h}}\Phi_{k^{\prime }}\left(\alpha_{i},\tau \right)h_{\alpha_{i}}^{*},$$

under the restriction of Equation (21). Here, $k^{\prime }$ is not necessarily any of $k_{1}$, $k_{2},\cdots$, $k_{M}$. Neither τ should be smaller than $N\tau_{0}$, where $\tau_{0}$ is the sampling interval and *N* number of time deviations. If we denote the minimum and maximum of Equation (25) as ${\underline{\sigma }}_{k^{\prime }}^{2}\left(\tau \right)$ and ${\bar{\sigma }}_{k^{\prime }}^{2}\left(\tau \right)$, respectively, $\left[{\underline{\sigma }}_{k^{\prime }}^{2}\left(\tau \right),{\bar{\sigma }}_{k^{\prime }}^{2}\left(\tau \right)\right]$ can be approximately considered as an 1−2ε confidence region of $\sigma_{k^{\prime }}^{2}$. This is the predictive model used in stochastic ONA.

## 4. Models for Discrete-Time Variances

In this section, we introduce a way to compute coefficient matrices Φ, B(ε) and B(1−ε) used in stochastic ONA. Because B(ε) and B(1−ε) can be computed from Φ and the inverse of the chi-square distribution function (7). Equation (7) is well-defined if the degrees of freedom ${\mathrm{EDF}}_{k}\left(\tau \right)$ is known. ${\mathrm{EDF}}_{k}\left(\tau \right)$, in turn, can be determined by Φ and $\mathrm{Var}\left[{\hat{\sigma }}_{k}^{2}\left(\tau \right)\right]$. Specifically, we: (i) formulate the influence of deterministic linear frequency drift on Allan (AVAR) and modified Allan (MVAR) variance; (ii) derive expressions for $\Phi_{k}\left(\alpha ,\tau \right)$ and $\mathrm{Var}\left[{\hat{\sigma }}_{k}^{2}\left(\tau \right)\right]$ of discrete-time Hadamard variance (HVAR). Computing the values of $\Phi_{k}\left(\alpha ,\tau \right)$ and $\mathrm{Var}\left[{\hat{\sigma }}_{k}^{2}\left(\tau \right)\right]$ for discrete-time AVAR, MVAR and HVAR is a daunting task, since we need to compute the gammafunctions O(mN) ($O\left(mN^{2}\right)$ for MVAR) times. We reduce the computation of gamma functions to three times per $f^{\alpha }$ noise in the end of this section.

### 4.1. Drift Model

To formulate the influences of deterministic linear frequency drift on AVAR and MVAR, we first assume the oscillator output signal to be contaminated by drift. Suppose its time derivations x[i] can be separated as:

$$x\left[i\right]=x^{\prime }\left[i\right]+a{\left(t+i\tau_{0}\right)}^{2},\;i=1,2,\cdots ,N,$$

where x[i] and $x^{\prime }\left[i\right]$ are discrete sampling of the continuous-time signals x(t) and $x^{\prime }\left(t\right)$, respectively. We also denote the AVAR and MVAR estimated from $x^{\prime }\left[i\right]$ as ${\hat{\sigma }}_{y}^{2}\left(x^{\prime },m\right)$ and Mod${\hat{\sigma }}_{y}^{2}\left(x^{\prime },m\right)$, respectively. Apparently,

$$E\left[{\hat{\sigma }}_{y}^{2}\left(x^{\prime },m\right)\right]=\sum_{i=1}^{N_{h}}\Phi_{\mathrm{AVAR}}\left(\alpha_{i},\tau \right)h_{\alpha_{i}}$$

and:

$$E\left[\mathrm{Mod}{\hat{\sigma }}_{y}^{2}\left(x^{\prime },m\right)\right]=\sum_{i=1}^{N_{h}}\Phi_{\mathrm{MVAR}}\left(\alpha_{i},\tau \right)h_{\alpha_{i}}.$$

Since $x^{\prime }\left[i\right]$ is unknown, AVAR and MVAR can only be measured from x[i]. We denote them as ${\hat{\sigma }}_{y}^{2}\left(x,m\right)$ and Mod${\hat{\sigma }}_{y}^{2}\left(x,m\right)$, respectively. It can be shown, from Equations (2) and (3), that:

(26) $$E\left[{\hat{\sigma }}_{y}^{2}\left(x,m\right)\right]=E\left[{\hat{\sigma }}_{y}^{2}\left(x^{\prime },m\right)\right]+2{\left(m\tau_{0}a\right)}^{2}$$

and:

(27) $$E\left[\mathrm{Mod}{\hat{\sigma }}_{y}^{2}\left(x,m\right)\right]=E\left[\mathrm{Mod}{\hat{\sigma }}_{y}^{2}\left(x^{\prime },m\right)\right]+2{\left(m\tau_{0}a\right)}^{2}.$$

In other words, the drift-free AVAR and MVAR can be divided from the influence of drift theoretically.

To predict long-term stability, the sign of *a* makes no difference. We can therein treat $a^{2}$ as a component of *h*. The column vector *h* of a rubidium frequency standard, for instance, is:

$${\left[\begin{array}{ccccccc} a^{2} & h_{2} & h_{1} & h_{0} & h_{-1} & h_{-2} & h_{-4} \end{array}\right]}^{T},$$

where $h_{\alpha }$, α=2, 1, 0, −1, −2 and −4 are noise intensity coefficients of white and flicker PM, white, flicker, random walk and random run FM, respectively. Accordingly, the *m*-th row of $\Phi_{k}$ and $B_{k}\left(\varepsilon \right)$ can be cast as:

(28) $$\left[\begin{array}{ccccccc} 2{\left(m\tau_{0}\right)}^{2} & \Phi_{\mathrm{AVAR}}\left(2,m\tau_{0}\right) & \Phi_{\mathrm{AVAR}}\left(1,m\tau_{0}\right) & \Phi_{\mathrm{AVAR}}\left(0,m\tau_{0}\right) & \Phi_{\mathrm{AVAR}}\left(-1,m\tau_{0}\right) & \Phi_{\mathrm{AVAR}}\left(-2,m\tau_{0}\right) & 0 \end{array}\right]$$

and:

(29) $${\left[\begin{array}{c} 2{\left(m\tau_{0}\right)}^{2}\cdot F^{-1}\left(\varepsilon \right)/{\left.{\mathrm{EDF}}_{\mathrm{AVAR}}\left(\tau \right)\right|}_{\alpha =2}\\ \Phi_{\mathrm{AVAR}}\left(2,m\tau_{0}\right)\cdot F^{-1}\left(\varepsilon \right)/{\left.{\mathrm{EDF}}_{\mathrm{AVAR}}\left(\tau \right)\right|}_{\alpha =2}\\ \Phi_{\mathrm{AVAR}}\left(1,m\tau_{0}\right)\cdot F^{-1}\left(\varepsilon \right)/{\left.{\mathrm{EDF}}_{\mathrm{AVAR}}\left(\tau \right)\right|}_{\alpha =1}\\ \vdots \\ \Phi_{\mathrm{AVAR}}\left(-2,m\tau_{0}\right)\cdot F^{-1}\left(\varepsilon \right)/{\left.{\mathrm{EDF}}_{\mathrm{AVAR}}\left(\tau \right)\right|}_{\alpha =-2}\\ 0 \end{array}\right]}^{T}$$

for AVAR, respectively. Here, we use the subscript α=2, 1, 0, −1, −2 or −4 to indicate the dominant PLN process; other PLN processes will be ignored in the computation of the inverse chi-square distribution function. While $\Phi_{\mathrm{AVAR}}\left(\alpha ,m\tau_{0}\right)$ is given in Equation (15) and $\mathrm{Var}\left[{\hat{\sigma }}_{\mathrm{AVAR}}^{2}\left(\tau \right)\right]$ in Equation (16) explicitly, it is a daunting task to compute $\Phi_{k}$ and $B_{k}\left(\varepsilon \right)$ from these equations. As we show at the end of this section, the computation can be greatly shortened by taking the properties of the gamma function into account. Especially, we can simplify Equation (15) in the case of GPS MCS clock prediction:

(30) $$E\left[{\hat{\sigma }}_{y}^{2}\left(x,m\right)\right]=2{\left(m\tau_{0}a\right)}^{2}+\frac{3q_{0}}{m^{2}\tau_{0}^{2}}+\frac{q_{1}}{m\tau_{0}^{2}}+\frac{q_{2}\left(2m^{2}+1\right)}{6m}.$$

On the other hand, the simplified expression for $\mathrm{Var}\left[{\hat{\sigma }}_{\mathrm{AVAR}}^{2}\left(\tau \right)\right]$ depends on the ratio of *m* to *N*. When m≤N/4,

(31) $$\mathrm{Var}\left[{\hat{\sigma }}_{y}^{2}\left(m\tau_{0}\right)\right]=\frac{q_{0}^{2}\left(35N-88m\right)}{{\left(N-2m\right)}^{2}{\left(m\tau_{0}\right)}^{4}}$$

for α=2,

(32) $$\mathrm{Var}\left[{\hat{\sigma }}_{y}^{2}\left(m\tau_{0}\right)\right]=q_{1}^{2}\frac{\frac{5}{3}N+\frac{4}{3}m^{2}N-\frac{7}{2}m-\frac{1}{2}m^{2}-3m^{3}}{4{\left(N-2m\right)}^{2}m^{3}\tau_{0}^{4}},$$

for α=0, and:

(33) $$\begin{array}{cc} \mathrm{Var}\left[{\hat{\sigma }}_{y}^{2}\left(m\tau_{0}\right)\right]= & q_{2}^{2}\frac{\frac{302}{35}m^{6}N+4m^{4}N+\frac{14}{5}m^{2}N+\frac{18}{7}N-\frac{101}{5}m^{7}-\frac{34}{5}m^{5}-\frac{19}{5}m^{3}-\frac{26}{5}m}{144{\left(N-2m\right)}^{2}m^{3}}. \end{array}$$

for α=−2. Simplified expressions for $\mathrm{Var}\left[{\hat{\sigma }}_{y}^{2}\left(m\tau_{0}\right)\right]$, m>N/4, will be given in Appendix B.

### 4.2. Hadamard Variance

To differentiate the influence of frequency drift from the random behavior of an oscillator, for example RWFM or RRFM, we can combine AVAR with some statistics, which are convergent for RRFM and free from drift. We choose HVAR among those statistics. In order to form coefficient matrices Φ, B(ε) and B(1−ε), we derive here $\Phi_{k}\left(\alpha ,\tau \right)$ and $\mathrm{Var}\left[{\hat{\sigma }}_{k}^{2}\left(\tau \right)\right]$ of discrete-time HVAR.

Since discrete-time PSD is not a direct sampling of the corresponding continuous-time PSD, the discrete-time HVAR is not a discrete sampling of the continuous function defined by Equation (5). On the other hand, the discrete-time symmetric two-time autocorrelation function is a direct sampling of its continuous counterpart (10). If we can recast the continuous-time HVAR as a combination of autocorrelation functions, the discrete-time HVAR can be derived from directly sampling the autocorrelation function. Equivalently, if the discrete-time HVAR can be expanded as a combination of autocorrelation functions, an explicit expression of the variance can be derived by replacing the autocorrelation functions with Equations (12)–(14).

From Equations (4) and (10),

(34) $$\begin{array}{cc} E\left[{\hat{\sigma }}_{z}^{2}\left(\tau \right)\right]= & \frac{1}{6\tau^{2}}\left[R_{x}\left(t+3\tau ,0\right)+9R_{x}\left(t+2\tau ,0\right)\right.+9R_{x}\left(t+\tau ,0\right)+R_{x}\left(t,0\right)\\ & -6R_{x}\left(t+\frac{5}{2}\tau ,\tau \right)+6R_{x}\left(t+2\tau ,2\tau \right)-18R_{x}\left(t+\frac{3}{2}\tau ,\tau \right)\\ & \left.-2R_{x}\left(t+\frac{3}{2}\tau ,3\tau \right)-6R_{x}\left(t+\frac{1}{2}\tau ,\tau \right)+6R_{x}\left(t+\tau ,2\tau \right)\right]. \end{array}$$

By substituting autocorrelation functions in the equation above with Equations (12) and (13), we attain:

(35) $$E\left[{\hat{\sigma }}_{z}^{2}\left(\tau \right)\right]=\left\{\begin{array}{cc} \frac{\sigma_{w_{\alpha }}^{2}\Gamma \left(\alpha -1\right){\left(\tau /\tau_{0}\right)}^{1-\alpha }}{\tau^{2}\Gamma \left(\alpha /2\right)\Gamma \left(1-\alpha /2\right)}\left(2^{2-\alpha }-5-3^{-\alpha }\right)+O\left(t^{-\alpha -5}\right), & \alpha \neq -1,\\ \frac{\sigma_{w_{\alpha }}^{2}}{3{\left(\tau_{0}\tau \right)}^{2}}\left[10\ln|\tau |-\ln\frac{64}{3}\right]+O\left(t^{-6}\right), & \alpha =-1. \end{array}\right.$$

Obviously, Equation (35) converges when α>−5. Because all of the power-law noises (PLN) mentioned before have a power index α>−5, Equation (35) holds for the problem discussed.

In addition, $t\geq \tau_{0}$, the sampling interval $\tau_{0}$ ranges from several minutes to days, and HVAR is approximately independent of *t* (We assume here that the random behaviors of an oscillator is unchanged. Otherwise, HVAR is either divergent or changes with time *t*). Hence, we replace the symmetric two-time autocorrelation function in Equation (35) with Equation (14). The expression for $\Phi_{k}\left(\alpha ,\tau \right)$ of discrete-time HVAR is therein derived:

(36) $$\begin{array}{c} \begin{array}{cc} \Phi_{z}\left(\alpha ,\tau \right)= & \frac{\Gamma (\alpha -1)}{6m^{2}{\left(2\pi \right)}^{\alpha }\tau_{0}^{\alpha +1}\Gamma \left(\alpha /2\right)\Gamma \left(1-\alpha /2\right)}\times \left[10\frac{\Gamma (1-\alpha /2)}{\Gamma (\alpha /2)}-\frac{15\Gamma (m+1-\alpha /2)}{\Gamma (m+\alpha /2)}\right.\\ & \left.+\frac{6\Gamma (2m+1-\alpha /2)}{\Gamma (2m+\alpha /2)}-\frac{\Gamma (3m+1-\alpha /2)}{\Gamma (3m+\alpha /2)}\right]. \end{array} \end{array}$$

autocorrelation functions in the equation above with Equations (12) and (13).

Likewise, we expand $\mathrm{Var}\left[{\hat{\sigma }}_{z}^{2}\left(\tau \right)\right]$ with the symmetric two-time autocorrelation functions:

(37) $$\begin{array}{c} \begin{array}{cc} \mathrm{Var}\left[{\hat{\sigma }}_{z}^{2}\left(\tau \right)\right]= & \sum_{\ell =-N+3m+1}^{N-3m-1}\frac{N-3m-\left|\ell \right|\tau_{0}}{18{\left(N-3m\right)}^{2}{\left(m\tau_{0}\right)}^{4}}\left\{R_{x}(t+3m\tau_{0},|\ell |\tau_{0})-3R_{x}(t+\frac{5}{2}m\tau_{0},|m+\ell |\tau_{0})\right.\\ & -3R_{x}(t+\frac{5}{2}m\tau_{0},|m-\ell |\tau_{0})+9R_{x}(t+2m\tau_{0},|\ell |\tau_{0})+3R_{x}(t+2m\tau_{0},|2m+\ell |\tau_{0})\\ & +3R_{x}(t+2m\tau_{0},|2m-\ell |\tau_{0})-R_{x}(t+\frac{3}{2}m\tau_{0},|3m+\ell |\tau_{0})-R_{x}(t+\frac{3}{2}m\tau_{0},|3m-\ell |\tau_{0})\\ & -9R_{x}(t+\frac{3}{2}m\tau_{0},|m+\ell |\tau_{0})-9R_{x}(t+\frac{3}{2}m\tau_{0},|m-\ell |\tau_{0})+9R_{x}(t+m\tau_{0},|\ell |\tau_{0})\\ & +3R_{x}(t+m\tau_{0},|2m+\ell |\tau_{0})+3R_{x}(t+m\tau_{0},|2m-\ell |\tau_{0})-3R_{x}(t+\frac{1}{2}m\tau_{0},|m+\ell |\tau_{0})\\ & {\left.-3R_{x}(t+\frac{1}{2}m\tau_{0},|m-\ell |\tau_{0})+R_{x}\left(t,|\ell \right|\tau_{0})\right\}}^{2} \end{array} \end{array}$$

by assuming the third order differences of x(t),

$$\left[x(t+3m)-3x(t+m)+3x(t+m)-x(t)\right]/\tau ,$$

*m* fixed, are normally distributed. It is easy to see that Equation (37) holds for α>−5 after substituting the autocorrelation functions in the above equation with Equations (12) and (13). Furthermore, $\mathrm{Var}\left[{\hat{\sigma }}_{z}^{2}\left(\tau \right)\right]$ is approximately independent of the parameter *t*. Therefore, we replace the autocorrelation functions with Equation (14). $\mathrm{Var}\left[{\hat{\sigma }}_{k}^{2}\left(\tau \right)\right]$ of HVAR is therein cast as

(38) $$\begin{array}{cc} \mathrm{Var}\left[{\hat{\sigma }}_{z}^{2}\left(\tau \right)\right]= & \frac{h_{\alpha }^{2}\Gamma^{2}\left(\alpha -1\right)\sin^{2}\left(\alpha \pi /2\right)}{2{\left(2\pi \tau_{0}\right)}^{2\alpha +2}\left(N-3m\right)m^{4}}\times \sum_{\ell =-N+3m+1}^{N-3m-1}\frac{N-3m-|\ell |}{N-3m}\\ & \times \left\{\frac{20\Gamma (|\ell |+1-\alpha /2)}{3\Gamma (|\ell |+\alpha /2)}-\frac{5\Gamma (|m+\ell |+1-\alpha /2)}{\Gamma (|m+\ell |+\alpha /2)}\right.\\ & -\frac{5\Gamma (|m-\ell |+1-\alpha /2)}{\Gamma (|m-\ell |+\alpha /2)}+\frac{2\Gamma (|2m+\ell |+1-\alpha /2)}{\Gamma (|2m+\ell |+\alpha /2)}\\ & +\frac{2\Gamma (|2m-\ell |+1-\alpha /2)}{\Gamma (|2m-\ell |+\alpha /2)}-\frac{\Gamma (|3m+\ell |+1-\alpha /2)}{3\Gamma (|3m+\ell |+\alpha /2)}\\ & {\left.-\frac{\Gamma (|3m-\ell |+1-\alpha /2)}{3\Gamma (|3m-\ell |+\alpha /2)}\right\}}^{2}. \end{array}$$

By then, the coefficient matrices Φ, B(ε) and B(1−ε) in stochastic ONA can be constructed in the following way:

$$\Phi =\left[\begin{array}{c} \Phi_{\mathrm{AVAR}}\\ \Phi_{\mathrm{HVAR}} \end{array}\right],\;B\left(\varepsilon \right)=\left[\begin{array}{c} B_{\mathrm{AVAR}}\left(\varepsilon \right)\\ B_{\mathrm{HVAR}}\left(\varepsilon \right) \end{array}\right],\;B\left(1-\varepsilon \right)=\left[\begin{array}{c} B_{\mathrm{AVAR}}\left(1-\varepsilon \right)\\ B_{\mathrm{HVAR}}\left(1-\varepsilon \right) \end{array}\right],$$

where $\Phi_{\mathrm{AVAR}}$ is defined in Equation (28), $B_{\mathrm{AVAR}}\left(\varepsilon \right)$ and $B_{\mathrm{AVAR}}\left(1-\varepsilon \right)$ in Equation (29). The *m*-th rows of $\Phi_{\mathrm{HVAR}}$ and $B_{\mathrm{HVAR}}\left(\varepsilon \right)$ are defined as:

(39) $$\left[\begin{array}{ccccc} 0 & \Phi_{\mathrm{HVAR}}\left(2,m\tau_{0}\right) & \cdots & \Phi_{\mathrm{HVAR}}\left(-2,m\tau_{0}\right) & \Phi_{\mathrm{HVAR}}\left(-4,m\tau_{0}\right) \end{array}\right]$$

and:

(40) $${\left[\begin{array}{c} 0\\ \Phi_{\mathrm{HVAR}}\left(2,m\tau_{0}\right)\cdot F^{-1}\left(\varepsilon \right)/{\left.{\mathrm{EDF}}_{\mathrm{HVAR}}\left(\tau \right)\right|}_{\alpha =2}\\ \Phi_{\mathrm{HVAR}}\left(1,m\tau_{0}\right)\cdot F^{-1}\left(\varepsilon \right)/{\left.{\mathrm{EDF}}_{\mathrm{HVAR}}\left(\tau \right)\right|}_{\alpha =1}\\ \vdots \\ \Phi_{\mathrm{HVAR}}\left(-2,m\tau_{0}\right)\cdot F^{-1}\left(\varepsilon \right)/{\left.{\mathrm{EDF}}_{\mathrm{HVAR}}\left(\tau \right)\right|}_{\alpha =-2}\\ \Phi_{\mathrm{HVAR}}\left(-4,m\tau_{0}\right)\cdot F^{-1}\left(\varepsilon \right)/{\left.{\mathrm{EDF}}_{\mathrm{HVAR}}\left(\tau \right)\right|}_{\alpha =-4} \end{array}\right]}^{T},$$

respectively. When the time series contains random run FM, $h_{-4}\neq 0$. While AVAR does not converge for α=−4 PLN, the noise process has little influence on short-term AVAR estimated from real data. In such a case, the inconsistency between Equations (29) and (40) will be treated as ‘violations’ by the optimization problem (23). The unformulated influence of α=−4 PLN in Equation (29) will be smoothed out by Equation (24).

In GPS MCS clock prediction, only PLN of α=2, 0, −2 and −4 are considered. In such a case, the flicker noise components in Φ, B(ε) and B(1−ε) should be removed. Furthermore, the remaining components can be computed using the following simplified expression:

(41) $$E\left[{\hat{\sigma }}_{z}^{2}\left(\tau \right)\right]=\frac{10q_{0}}{3m^{2}\tau_{0}^{2}}+\frac{q_{1}}{m\tau_{0}^{2}}+\frac{q_{2}}{6m}\left(m^{2}+1\right)+\frac{q_{3}\tau_{0}^{2}}{120m}\left(11m^{4}+5m^{2}-4\right).$$

If, in addition, m≤N/6,

(42) $$\mathrm{Var}\left[{\hat{\sigma }}_{z}^{2}\left(\tau \right)\right]=\frac{\left(154N-562m\right)q_{0}^{2}}{3{\left(N-3m\right)}^{2}{\left(m\tau_{0}\right)}^{4}}$$

for α=2,

(43) $$\mathrm{Var}\left[{\hat{\sigma }}_{z}^{2}\left(\tau \right)\right]=q_{1}^{2}\frac{56m^{3}N+84mN-204m^{4}-288m^{2}}{144{\left(N-3m\right)}^{2}{\left(m\tau_{0}\right)}^{4}}$$

for α=0,

(44) $$\mathrm{Var}\left[{\hat{\sigma }}_{z}^{2}\left(\tau \right)\right]=q_{2}^{2}\frac{62m^{6}N+92m^{4}N+98m^{2}N+108N-\frac{1557}{7}m^{7}-312m^{5}-309m^{3}-\frac{2496}{7}m}{4320{\left(N-3m\right)}^{2}m^{3}}$$

for α=−2, and:

(45) $$\begin{array}{cc} \mathrm{Var}\left[{\hat{\sigma }}_{z}^{2}\left(\tau \right)\right]= & \frac{\tau_{0}^{4}q_{3}^{2}}{4147200{\left(N-3m\right)}^{2}m^{3}}\left(\frac{2620708}{231}m^{10}N\right.+\frac{16180}{3}m^{8}N+3844m^{6}N\\ & +\frac{63940}{21}m^{4}N+\frac{7664}{3}m^{2}N+\frac{28800}{11}N-\frac{2979934}{77}m^{11}-\frac{104194}{7}m^{9}\\ & -\frac{70662}{7}m^{7}-\frac{49702}{7}m^{5}-\frac{50744}{7}m^{3}\left.-\frac{644544}{77}m\right). \end{array}$$

for α=−4. Expressions of $\mathrm{Var}\left[{\hat{\sigma }}_{z}^{2}\left(\tau \right)\right]$ for m>N/6 will be given in Appendix B.

### 4.3. Quick Computation of Discrete-Time Variances

Although for Equations (36) and (38), Walter’s characterizations of AVAR and HVAR holds for real α values, they produce heavy computational burdens. If their computational complexity is represented by the evaluation of Gamma functions, then, for given *m*, the complexity of Equations (15), (16), (36) and (38) is O(N) and $O\left(mN\right)$ for Walter’s characterization of MVAR. Here, we describe a method to reduce the computation complexity to three.

In order to estimate the values of Equations (15), (16), (36) and (38), we define an *N*-dimensional column vector $b_{\Gamma }$. The *i*-th component of $b_{\Gamma }$ is:

(46) $$b_{\Gamma }\left(i\right)=\sin\left(\frac{\alpha }{2}\pi \right)\frac{\Gamma \left(i-\frac{\alpha }{2}\right)\Gamma \left(\alpha -1\right)}{\Gamma \left(i-1+\frac{\alpha }{2}\right)}.$$

From the properties of the gamma function, we recast Equations (15), (16), (36) and (38) as functions of $b_{\Gamma }$. For instance,

$$\Phi_{\mathrm{AVAR}}\left(\alpha ,\tau \right)=\frac{3b_{\Gamma }\left(1\right)-4b_{\Gamma }\left(m+1\right)+b_{\Gamma }\left(2m+1\right)}{m^{2}{\left(2\pi \tau_{0}\right)}^{\alpha +1}}$$

and:

$$\mathrm{Var}\left[{\hat{\sigma }}_{y}^{2}\left(\tau \right)\right]=\sum_{\ell =-N+2m+1}^{N-2m-1}\frac{N-2m-|\ell |}{2}{\left[\sum_{i=-2}^{2}h_{\alpha }\frac{2\left(3-|i|\right)b_{\Gamma }\left(|im+\ell |+1\right)}{{\left(2\pi \tau_{0}\right)}^{\alpha +1}\left(N-2m\right)m^{2}}\right]}^{2}.$$

It is obvious that:

|im+ℓ|≤N−1,−N+2m+1≤ℓ≤N−2m−1.

On the other hand, for any 0≤j≤N−1, there exists *ℓ* such that:

|im+ℓ|=j,

for some −2≤i≤2. Hence, the auxiliary parameter $b_{\Gamma }$ is both sufficient and necessary in the computation of Equations (15), (16), (36) and (38).

To calculate the values of $b_{\Gamma }$, we start by searching for the least positive $i_{0}$ such that:

$$i-\frac{\alpha }{2}\geq 1$$

and:

$$i-1+\frac{\alpha }{2}\geq 1.$$

Then, we compute the value of $b_{\Gamma }\left(i_{0}\right)$:

$$b_{\Gamma }\left(i_{0}\right)={\left(-1\right)}^{\left\lfloor \alpha /2\right\rfloor }\sin\left(\frac{\alpha }{2}\pi -\left\lfloor \frac{\alpha }{2}\right\rfloor \pi \right)\frac{\Gamma \left(i_{0}-\frac{\alpha }{2}\right)\Gamma \left(\alpha -1\right)}{\Gamma \left(i_{0}-1+\frac{\alpha }{2}\right)}$$

Other components of $b_{\Gamma }$ can be estimated recursively: Given the value of $b_{\Gamma }\left(i\right)$:

- if $b_{\Gamma }\left(i-1\right)$ is unknown,
  $$b_{\Gamma }\left(i-1\right)=\frac{i-2+\alpha /2}{i-1-\alpha /2}b_{\Gamma }\left(i\right);$$
- if $b_{\Gamma }\left(i+1\right)$ is unknown,
  $$b_{\Gamma }\left(i+1\right)=\frac{i-\alpha /2}{i-1+\alpha /2}b_{\Gamma }\left(i\right).$$

By using the auxiliary vector $b_{\Gamma }$, we reduce the calculations of gamma functions to three times per $f^{\alpha }$ noise.

## 5. Results and Discussion

To test the method proposed in this article, we predict τ=15-day frequency stabilities of GPS onboard clocks. The predictions are made based on 14-day GPS precise clock data provided by IGS (International GNSS Service). The IGS timescale is selected as the reference clock. For comparison, we also estimate variances from 42–60-day measured data. It should be noted that the method discussed in this paper assumes power-law processes and deterministic frequency drift being the major sources of time-series data. Analysis of all thirty-two satellites shows that the method fails when period behaviors have a strong influence on input variances. In this section, only the predictions of GPS SVN. 45 and 41 satellite rubidium clock frequency stabilities are chosen as representative. This is because:

- To test the modified stochastic ONA method, a strong presentation of deterministic frequency drift should be seen. In the real data test of [7], frequency drift does not have significant influence on the behaviors of some onboard rubidium frequency standards within 168 days. Such data cannot test the capability of stochastic ONA in predicting drift contaminated stabilities.
- The oscillator should be somehow well-modeled. If the dominant variation of the frequency standard has not been model and it has major influence on frequency stability estimates, stochastic ONA will not function properly. For instance, stochastic ONA fails to predict the long-term AVAR and HVAR from MJD.52437.0-52451.0 GPS SVN.36 (PRN.06) precise clock data. AVAR and HVAR estimated from the data imply strong periodic behaviors.

### 5.1. SVN.45

**Figure 1:**
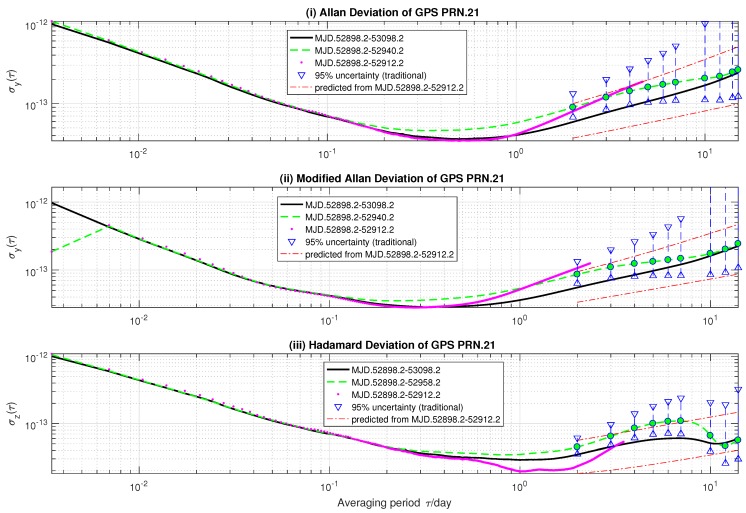
Estimates and prediction of GPS SVN.45 (PRN.21) rubidium clock frequency stability.

The prediction of GPS SVN.45 onboard clock long-term stability shows how stochastic ONA behaves when it cannot distinguish RWFM from frequency drift. As shown in Figure 1, we use (i) AVAR, (ii) MVAR and (iii) HVAR estimated from 84-day GPS SVN.45 rubidium precise clock data (‘—’) as the reference. Although a linear frequency drift was removed before the estimation, the variances are not free from drift. The predictions (‘–·–’) made by stochastic ONA are based on variances estimated from the first 14 days of the time deviations (‘⋯’). Since we set ε = 0.025 in the computation, the predicted confidence interval of long-term variance can be seen to have a 95%-confidence level. For comparison, we also estimate (i) AVAR and (ii) MVAR from the first 42-day (‘– –’) and (iii) HVAR from the first 60-day (‘– –’) measured data. By assuming RWFM (α=−2) as the dominant noise, 95%-confidence regions of there variances (‘▹–∘–◃’) are computed in the following way:

(47) $$\left[\frac{{\mathrm{EDF}}_{k^{\prime }}\left(\tau \right)}{F^{-1}\left(97.5\%\right)}{\hat{\sigma }}_{k^{\prime }}^{2}\left(\tau \right),\;\frac{{\mathrm{EDF}}_{k^{\prime }}\left(\tau \right)}{F^{-1}\left(2.5\%\right)}{\hat{\sigma }}_{k^{\prime }}^{2}\left(\tau \right)\right].$$

It can be seen from Figure 1 that 0.1–1-day AVAR and HVAR estimated from 14-day measured data are less than the referenced variances. On the other hand, τ>1-day AVAR and HVAR estimated from 14 days are much larger than the reference. This will be interpreted as a weaker FLFM and stronger RWFM by the conventional oscillator noise analyzer. Stochastic ONA attributes the fluctuations to RWFM, because RWFM has much larger confidence regions at long-term averaging time. For example, the 1152-th (τ = 4/day) rows of $\Phi_{y}$ and $B_{y}\left(\varepsilon =0.025\right)$ have the following approximate values:

$${\left[\begin{array}{c} 2.39\times 10^{11}\\ 1.06\times 10^{-15}\\ 5.60\times 10^{-12}\\ 1.45\times 10^{-6}\\ 1.40\\ 2.27\times 10^{6}\\ 0 \end{array}\right]}^{T}\;\mathrm{and}\;{\left[\begin{array}{c} 2.14\times 10^{11}\\ 9.81\times 10^{-16}\\ 3.20\times 10^{-12}\\ 1.30\times 10^{-7}\\ 6.21\times 10^{-2}\\ 4.05\times 10^{4}\\ 0 \end{array}\right]}^{T},$$

respectively. Consequently, the predicted lower and upper bounds of long-term variance are almost parallel to the references. Stochastic ONA cannot find any *h* that holds for Equation (21), so it has to make a trade-off between 0.1–1-day and τ>1-day input variances: while the former lead to a smaller RWFM noise level, the latter indicate a larger one. Although the predicted confidence regions are greater than those estimated from 42-day measured data for averaging time of τ≤10 days, they are consistent with the referenced variances. However, the confidence regions of variances estimated from 42-day data, by contrast, do not encompass all referenced variances. For instance, τ=1- and 2-day referenced AVAR and MVAR, and τ = 2∼7-day HVAR are not included in the confidence regions calculated from 42-day clock data.

### 5.2. SVN.41

**Figure 2:**
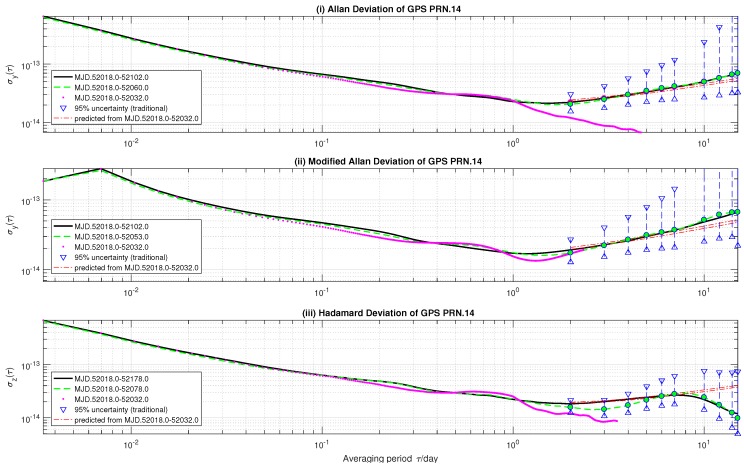
Estimates and prediction of GPS SVN.41 (PRN.14) rubidium clock frequency stability.

When stochastic ONA does find an *h* that holds for Equation (21), it predicts long-term variances with narrow confidence regions. This can be seen in Figure 2. The input variances (‘⋯’) of stochastic ONA are estimated from GPS SVN.41 rubidium clock time deviations from MJD.52018.0–MJD.52032.0. Stochastic ONA predicts 2-day ≤τ≤15-day AVAR, MVAR and HVAR (‘–·–’) based on these variances with ε = 0.025. The referenced (i) AVAR and (ii) MVAR are measured from 84-day (‘—’); and (iii) HVAR 160-day (‘—’) time deviations of the same clock. For comparison, we also estimate (i) AVAR from 42-day (‘– –’), (ii) MVAR from 45-day (‘– –’) and (iii) HVAR from 60-day (‘– –’) measured data. Their 95%-confidence regions (‘▹–∘–◃’) are computed by assuming RWFM as the dominant noise.

In Figure 2, only the τ≤7-day referenced variances are included in the confidence intervals predicted by stochastic ONA. This phenomenon can be explained by the behavior of input variances. By comparing Figure 2 with Figure 1, it is easy to see that the 0.1-day ≤τ≤1-day frequency stabilities of the GPS SVN.41 onboard clock do not vibrate so fiercely as GPS SVN.45’s. On the other hand, the former has a tail tens of times smaller than the referenced. As discussed in the previous subsection, stochastic ONA tends to attribute the fluctuations at long-term averaging time to RWFM. Stochastic ONA finds an *h* that holds for Equation (21). In this case, stochastic ONA fits the input variances using the inequality-bounded least-square model (17). However, the least square criterion (17) is designed for Gaussian distributions. In addition, the existence of some 1−2ε confidence regions holding for input variances does not mean no violation to the theoretical 1−2ε confidence intervals. Consequently, as shown in Figure 2, stochastic ONA underestimates the influence of frequency drift and overestimates the noise level of RWFM. Despite their compactness, only τ≤ one-week referenced variances are included in the predicted regions. On the other hand, all referenced variances of the SVN.45 satellite clock are included in the regions predicted without using the least-square criterion.

## 6. Conclusions

In this article, we discussed the prediction of long-term stability with the presentation of deterministic linear frequency drift. The fundamental theory of time stability analysis and the influences of discrete sampling are first revisited. Based on these theories, we construct a method called stochastic ONA. Stochastic ONA extends the capability of conventional oscillator noise analysis to the prediction of long-term frequency stability. By then, we introduce methods to model long-term variances contaminated by frequency drift. Specifically, we: (i) formulated the influence of frequency drift on Allan (AVAR) and modified Allan (MVAR) variances; (ii) derived expressions for discrete Hadamard variance (HVAR); (iii) simplified the formulations for the case of GPS MCS (master control station) clock prediction; and (iv) introduce a method that reduces the computational complexity of Walter’s characterization of AVAR and MVAR.

To test stochastic ONA and the model, we predict τ≤15-day AVAR, MVAR and HVAR based on 14-day GPS precise clock data. Due to limited space, we choose the result of GPS SVN 45 and 41 as representatives:

- For the SVN.45 satellite clock, stochastic ONA cannot find a set of noise intensity coefficients for which Equation (21) holds for input variances. In such a case, stochastic ONA predicts long-term stabilities based on Equation (23). The criterion (23) takes the probability distributions of input variances into account and produces robust results. All the referenced variances are included in the predicted confidence regions. On the other hand, τ=1- and 2-day referenced AVAR and MVAR, and τ = 2∼7-day HVAR are not included in the 95%-confidence regions estimated from 42-day clock data.
- For the SVN.41 onboard clock, stochastic ONA does find noise intensity coefficient values that hold for the probabilistic constraints (21). In this case, it predicts long-term stability of the satellite frequency standard based on the least square criterion (17). Despite the compactness of predicted confidence intervals, only τ≤7-day referenced variances are included in these regions. Specifically, τ>7-day referenced AVAR and MVAR are greater than the predicted ones, while the τ>7-day referenced HVAR is smaller than the predictions. This suggests an overestimation of RWFM noise level and underestimation of frequency drift. Nevertheless, the inconsistency may be interpreted as the inappropriate power-law model used in this paper.

In summary, the method introduced in this paper can predict long-term stability superimposed with influences of frequency drift. Criterion (23) takes the probability distributions of input variances into account by assuming the majority of input variances within their 1−2ε confidence regions. The predictions made therein have large uncertainty, but are robust. In contrast, the least square criterion (17) assumes non-existing symmetric distributions of input variances, which reduce both the uncertainty and robustness of the result. We should find an alternative for the least square criterion in a future study.

## Abbreviations

The following abbreviations are used in this manuscript:

AVAR	Allan variance
EDF	Equivalence degree of freedom
FLFM	Flicker frequency modulation
FLPM	Flicker phase modulation
GNSS	Global navigation satellite system
GPS	Global positioning system
HVAR	Hadamard variance
MCS	Master control station
MVAR	Modified Allan variance
ONA	Oscillator noise analysis
RRFM	Random run frequency modulation
RWFM	Random walk frequency modulation
PLN	Power-law noise
PRN	Pseudo-random noise
PSD	Power spectral distribution or power spectrum density
SVN	Space vehicle numbers
TAI	International Atomic Time
WHFM	White frequency modulation
WHPM	White phase modulation

## Appendix A. Convex Optimization Techniques

We solve the stochastic ONA models described in this paper using a primal-dual interior-point algorithm. Its foundation is the theory of convex optimization. In this Appendix, relevant concepts and techniques will be reviewed.

### Appendix A.1. ℓ_1_ Norm and Compressive Sensing

We define the object function in Equation (23) using the $\ell_{1}$-norm:

$$f_{0}\left(h,\mu ,\nu \right)={∥\mu ∥}_{1}+{∥\nu ∥}_{1}.$$

Since both μ and ν are positive,

$$f_{0}\left(h,\mu ,\nu \right)=\sum_{i=1}^{\sum_{j=1}^{M}m_{j}}\left(\mu_{i}+\nu_{i}\right).$$

It has been shown that when the $\ell_{1}$-norm is applied to the optimization variable, and

dim(h,μ,ν)>dim(σ)

estimates of the optima are approximately sparse [20]. Here, dim(σ) returns the dimension of σ. There is a high probability that the solution is stable and unique. The number:

dim(h,μ,ν)=dim(h)+dim(μ)+dim(ν).

makes little difference to the solutions. Instead, (h,μ,ν) should be less than Cdim(σ), where C is a constant given in [21]. Such kinds of problems are called compressive sensing and can be solved by the convex optimization techniques described below.

### Appendix A.2. Solving Inequality Constrained Optimization Problems

Equations (20), (23) and (25) are all convex optimization problems, because both sets defined by Equation (21) and the inequality constraints of Equation (23) are intersections of hyperplanes. That is, they are convex sets. In addition, the object functions defined in Equations (20), (23) and (25) are convex since they are either linear or quadratic with the semi-positive Hessian.

If we denote the object functions of Equations (20), (23) and (25) as $f_{0}\left(s\right)$, where s=h or (h,μ,ν), and the inequality constraints as:

$$f_{i}\left(s\right)\leq 0,\;i=1,2,\cdots ,l,$$

where $l=3\sum_{j=1}^{M}m_{j}$ or $6\sum_{j=1}^{M}m_{j}$. The (Lagrange) dual problem of Equation (20), (23) and (25) is defined as:

(A1) $$\begin{array}{c} \begin{array}{ccc} \mathrm{maximize} & \; & L(\lambda ),\\ \text{subject to} & \; & \lambda \geq 0. \end{array} \end{array}$$

where λ is an *l*-dimensional real column vector, L(λ) dual function of the original problem:

(A2) $$L\left(\lambda \right):=\underset{(s)\in D}{\inf}\left(r(s,\lambda )\right),$$

the infimum function $\inf_{(s)\in D}\left(r(s,\lambda )\right)$ returns the maximum value of A, A is the set of all real values less than r(s,λ) for s∈D and fixed λ. r(s,λ) is the corresponding Lagrangian,

(A3) $$r\left(s,\lambda \right)=f_{0}\left(s\right)+\sum_{i=1}^{l}\lambda_{i}f_{i}\left(s\right).$$

Then, $L\left(\lambda \right)\leq f_{0}\left(s\right)$. If, in addition, all inequalities $f_{i}\left(s\right)\leq 0$, i=1, …, *l*, are strict, and we denote the optimum of $f_{0}\left(s\right)$ and L(λ) as $s^{*}$ and $\lambda^{*}$, respectively,

$$f_{0}\left(s^{*}\right)=L\left(\lambda^{*}\right).$$

Since the object function $f_{0}\left(s\right)$ defined by Equations (20), (23) and (25) and their dual L(λ) are differentiable,

(A4) $${\left.\left[\frac{\partial r(s,\lambda )}{\partial s}\;\frac{\partial r(s,\lambda )}{\partial \lambda }\right]\right|}_{\left(s,\lambda \right)=\left(s^{*},\lambda^{*}\right)}=0.$$

This is the famous Karush–Kuhn–Tucker (KKT) conditions.

To solve the inequality constraints, we replace $f_{i}\left(s\right)$ with the interior barriers:

$$I_{i}\left(s\right)=-\left(1/\eta \right)\log\left(-f_{i}\left(s\right)\right),\;i=1,\cdots ,l,$$

where log is the natural logarithm function and η an arbitrary positive real number. These barriers will be used as ‘penalties’ in the cost function. Specifically, we substitute the original object function $f_{0}\left(s\right)$ by:

(A5) $$f_{0}\left(s\right)-\sum_{i=1}^{m_{1}}\left(1/\eta \right)\log\left(-f_{i}\left(s\right)\right).$$

Since the inequality constraints become strict after taking the interior barriers, the KKT conditions are both sufficient and necessary for optima of Equation (A5). We therein solve Equation (A5) in the following Newtonian framework:

- $\left(s^{0},\lambda^{0}\right)$ is an arbitrary initial point of the original and dual problem
- for k=0, 1, ⋯
- solve the Newton step $\left(\Delta s^{k},\Delta \lambda^{k}\right)$ from:
  (A6) $$R\left[\begin{array}{c} \Delta s^{k}\\ \Delta \lambda^{k} \end{array}\right]=\left[\begin{array}{c} -\partial f_{0}\left(s\right)/\partial s-DF{\left(s\right)}^{T}\lambda \\ \mathrm{diag}(\lambda )F(s)+(1/\eta )1 \end{array}\right]$$
  at the point $\left(s,\lambda \right)=\left(h^{k},s^{k},\lambda^{k}\right)$, where:
  $$F\left(s\right)=\left[\begin{array}{cccc} f_{1}\left(s\right) & f_{2}\left(s\right) & \cdots & f_{l}\left(s\right), \end{array}\right]$$
  **1** is an *l*-dimensional column vector whose elements are one,
  $$R=\left[\begin{array}{cc} \nabla^{2}f_{0}\left(s\right)+\sum_{i=1}^{l}\lambda_{i}\nabla^{2}f_{i}\left(h,s\right) & DF{\left(s\right)}^{T}\\ \mathrm{diag}(\lambda )DF(s) & \mathrm{diag}\left(F(s)\right) \end{array}\right]$$
  diag(λ) returns a square diagonal matrix with the elements of vector λ on the main diagonal, and the operator $\nabla^{2}$ returns the Hessian matrix of the operand at its right-hand side; for example, for $f_{0}\left(h,s\right)$ defined in Equation (17), the *i*-th row and *j*-th column component of $\nabla^{2}f_{0}\left(s\right)$ is:
  $$\nabla^{2}f_{0}\left(s\right)\left[ij\right]=\frac{\partial^{2}f_{0}\left(s\right)}{\partial h_{\alpha_{i}}\partial h_{\alpha_{j}}}=\Phi^{T}Q\Phi \left[ij\right],$$
  $\Phi^{T}Q\Phi \left[ij\right]$ is the *i*-th row and *j*-th column component of $\Phi^{T}Q\Phi$, $\lambda_{i}$ the *i*-th component of λ, DF(s) the derivative matrix of F(s), whose *i*-th row *j*-th column component is:
  $$DF\left(s\right)\left[ij\right]=\frac{\partial f_{i}\left(s\right)}{\partial h_{\alpha_{j}}},$$
- set
  $$\left(h^{k+1},s^{k+1},\lambda^{k+1}\right)\leftarrow \left(h^{k},s^{k},\lambda^{k}\right)+\psi \left(\Delta h^{k},\Delta s^{k},\Delta \lambda^{k}\right)$$
  choosing β so that $f_{i}\left(s\right)<0$, i=1, ⋯, $m_{1}$, and λ≥0 [22], where 0<ψ≤1, ψ can be determined using a backtracking line search described in [23]. When ψ=1, the primal-dual interior-point algorithm is based on the pure Newton method.

If the difference between $f_{0}\left(s^{k}\right)$ and $r(s^{k},\lambda^{k})$ grows when *k* increases, from [24], we label Equation (A5) as ‘infeasible’, and change the model according to the description of Section 3. Otherwise, we increase *t* in Equation (A5) and use the result of the previous iteration as the initial point $\left(s^{0},\lambda^{0}\right)$ in th current iteration.

## Appendix B. Simplified Formulations

Suppose we estimate AVAR ${\hat{\sigma }}_{y}^{2}\left(m\tau_{0}\right)$ and HVAR ${\hat{\sigma }}_{z}^{2}\left(m\tau_{0}\right)$ from time deviations x[i], i=1, 2,⋯, and *N*, of a frequency standard. In Section 4, we give the expressions $\mathrm{Var}\left[{\hat{\sigma }}_{k}^{2}\left(m\tau_{0}\right)\right]$ of AVAR for m≤N/4 and HVAR for m≤N/6. Here, expressions of $\mathrm{Var}\left[{\hat{\sigma }}_{k}^{2}\left(m\tau_{0}\right)\right]$ for other situations will be given.

When N/3≤m<N/2,

$$\mathrm{Var}\left[{\hat{\sigma }}_{y}^{2}\left(m\tau_{0}\right)\right]=\frac{18q_{0}^{2}}{\left(N-2m\right){\left(m\tau_{0}\right)}^{4}}$$

for α=−2,

$$\mathrm{Var}\left[{\hat{\sigma }}_{y}^{2}\left(m\tau_{0}\right)\right]=q_{1}^{2}\frac{\frac{3}{4}N^{4}-8mN^{3}+32m^{2}N^{2}-\frac{3}{4}N^{2}-56m^{3}N+5mN+36m^{4}-7m^{2}}{4{\left(N-2m\right)}^{2}{\left(m\tau_{0}\right)}^{4}}$$

for α=0, and:

$$\begin{array}{cc} \mathrm{Var}\left[{\hat{\sigma }}_{y}^{2}\left(m\tau_{0}\right)\right]= & \frac{q_{2}^{2}}{96{\left(N-2m\right)}^{2}m^{4}}\left(\frac{3}{14}N^{8}-\frac{32}{7}mN^{7}+\frac{208}{5}m^{2}N^{6}-\frac{9}{5}N^{6}-\frac{1048}{5}m^{3}N^{5}\right.\\ & +\frac{144}{5}mN^{5}+\frac{1904}{3}m^{4}N^{4}-\frac{560}{3}m^{2}N^{4}+\frac{9}{2}N^{4}-\frac{3520}{3}m^{5}N^{3}\\ & +\frac{1864}{3}m^{3}N^{3}-48mN^{3}+\frac{3872}{3}m^{6}N^{2}-1104m^{4}N^{2}+\frac{2816}{15}m^{2}N^{2}\\ & -\frac{102}{35}N^{2}-\frac{27392}{35}m^{7}N+\frac{14624}{15}m^{5}N-\frac{4688}{15}m^{3}N+\frac{88}{5}mN\\ & +\frac{22016}{105}m^{8}-\frac{4864}{15}m^{6}+\frac{2792}{15}m^{4}-\left.\frac{824}{35}m^{2}\right) \end{array}$$

for α=−2.

When N/4≤m<N/3,

$$\mathrm{Var}\left[{\hat{\sigma }}_{y}^{2}\left(m\tau_{0}\right)\right]=\frac{2q_{0}^{2}\left(17N-42m\right)}{{\left(N-2m\right)}^{2}{\left(m\tau_{0}\right)}^{4}}$$

and:

$$\mathrm{Var}\left[{\hat{\sigma }}_{z}^{2}\left(m\tau_{0}\right)\right]=\frac{200q_{0}^{2}}{9\left(N-3m\right){\left(m\tau_{0}\right)}^{4}}$$

for α=2,

$$\begin{array}{cc} \mathrm{Var}\left[{\hat{\sigma }}_{y}^{2}\left(m\tau_{0}\right)\right]= & \frac{q_{1}^{2}}{8{\left(N-2m\right)}^{2}{\left(m\tau_{0}\right)}^{4}}\left(\frac{1}{6}N^{4}-2mN^{3}+8m^{2}N^{2}-mN^{2}\right.\\ & \left.-\frac{1}{6}N^{2}-8m^{3}N+8m^{2}N+5mN-6m^{4}-17m^{3}-11m^{2}\right) \end{array}$$

and:

$$\begin{array}{cc} \mathrm{Var}\left[{\hat{\sigma }}_{z}^{2}\left(m\tau_{0}\right)\right]= & \frac{q_{1}^{2}}{144{\left(N-3m\right)}^{2}{\left(m\tau_{0}\right)}^{4}}\left(\frac{100}{3}N^{4}-480mN^{3}+2592m^{2}N^{2}\right.\\ & \left.-\frac{100}{3}N^{2}-6192m^{3}N+280mN+5508m^{4}-540m^{2}\right) \end{array}$$

for α=0,

$$\begin{array}{cc} \mathrm{Var}\left[{\hat{\sigma }}_{y}^{2}\left(m\tau_{0}\right)\right]= & \frac{q_{2}}{72m^{4}{\left(N-2m\right)}^{2}}\left(\frac{1}{56}N^{8}-\frac{4}{7}mN^{7}+8m^{2}N^{6}-\frac{3}{20}N^{6}-64m^{3}N^{5}+\frac{18}{5}mN^{5}\right.\\ & +320m^{4}N^{4}-36m^{2}N^{4}+\frac{3}{8}N^{4}-1024m^{5}N^{3}+192m^{3}N^{3}-6mN^{3}+2048m^{6}N^{2}\\ & -576m^{4}N^{2}+36m^{2}N^{2}-\frac{17}{70}N^{2}-\frac{81618}{35}m^{7}N+\frac{4628}{5}m^{5}N+\frac{40253}{35}m^{8}\\ & -\frac{3106}{5}m^{6}-\frac{466}{5}m^{3}N+\frac{158}{35}mN+\frac{461}{5}m^{4}-\left.\frac{318}{35}m^{2}\right) \end{array}$$

and:

$$\begin{array}{cc} \mathrm{Var}\left[{\hat{\sigma }}_{z}^{2}\left(m\tau_{0}\right)\right]= & \frac{q_{2}^{2}}{648{\left(N-3m\right)}^{2}m^{4}}\left(\frac{25}{28}N^{8}-\frac{180}{7}mN^{7}\right.+\frac{1602}{5}m^{2}N^{6}-\frac{15}{2}N^{6}-\frac{11271}{5}m^{3}N^{5}\\ & +162mN^{5}+\frac{19575}{2}m^{4}N^{4}-1440m^{2}N^{4}+\frac{75}{4}N^{4}-26838m^{5}N^{3}+6735m^{3}N^{3}\\ & -270mN^{3}+45369m^{6}N^{2}-\frac{34911}{2}m^{4}N^{2}+\frac{7218}{5}m^{2}N^{2}-\frac{85}{7}N^{2}-\frac{1513107}{35}m^{7}N\\ & +23733m^{5}N-\frac{16923}{5}m^{3}N+\frac{666}{7}mN+\frac{2490669}{140}m^{8}-13203m^{6}\\ & +\frac{58653}{20}m^{4}\left.-\frac{1233}{7}m^{2}\right) \end{array}$$

for α=−2, and:

$$\begin{array}{cc} \mathrm{Var}\left[{\hat{\sigma }}_{z}^{2}\left(m\tau_{0}\right)\right]= & \frac{\tau_{0}^{4}q_{3}^{2}}{103680{\left(N-3m\right)}^{2}m^{4}}\left(\frac{5}{66}N^{12}-\frac{36}{11}mN^{11}\right.+64m^{2}N^{10}-\frac{35}{18}N^{10}-\frac{2245}{3}m^{3}N^{9}\\ & +70mN^{9}+\frac{81495}{14}m^{4}N^{8}-\frac{15675}{14}m^{2}N^{8}+\frac{755}{42}N^{8}-\frac{221962}{7}m^{5}N^{7}+\frac{73260}{7}m^{3}N^{7}\\ & -\frac{3624}{7}mN^{7}+\frac{619836}{5}m^{6}N^{6}-63243m^{4}N^{6}+\frac{32199}{5}m^{2}N^{6}-\frac{145}{2}N^{6}-\frac{1752408}{5}m^{7}N^{5}\\ & +257948m^{5}N^{5}-\frac{225667}{5}m^{3}N^{5}+1566mN^{5}+\frac{1423227}{2}m^{8}N^{4}-718917m^{6}N^{4}\\ & +194724m^{4}N^{4}-\frac{27821}{2}m^{2}N^{4}+\frac{1120}{9}N^{4}-1013166m^{9}N^{3}+1352484m^{7}N^{3}\\ & -529228m^{5}N^{3}+\frac{194878}{3}m^{3}N^{3}-1792mN^{3}+\frac{4809249}{5}m^{10}N^{2}-\frac{3291663}{2}m^{8}N^{2}\\ & +\frac{4424766}{5}m^{6}N^{2}-\frac{2351637}{14}m^{4}N^{2}+\frac{66942}{7}m^{2}N^{2}-\frac{5240}{77}N^{2}-\frac{211070799}{385}m^{11}N\\ & +\frac{8206362}{9}m^{9}N-\frac{29175021}{35}m^{7}N+\frac{1595052}{7}m^{5}N-\frac{156444}{7}m^{3}N+\frac{3648}{7}mN\\ & +\frac{21992877}{154}m^{12}-\frac{5208975}{14}m^{10}+\frac{4756509}{14}m^{8}-\frac{1773297}{14}m^{6}+\frac{134982}{7}m^{4}\left.-\frac{73224}{77}m^{2}\right) \end{array}$$

for α=−4.

When N/5≤m<N/4,

$$\mathrm{Var}\left[{\hat{\sigma }}_{z}^{2}\left(m\tau_{0}\right)\right]=\frac{\left(425N-1500m\right)q_{0}^{2}}{9{\left(N-3m\right)}^{2}{\left(m\tau_{0}\right)}^{4}}$$

for α=2,

$$\mathrm{Var}\left[{\hat{\sigma }}_{z}^{2}\left(m\tau_{0}\right)\right]=q_{1}^{2}\frac{\frac{25}{12}N^{4}-40mN^{3}+288m^{2}N^{2}-\frac{25}{12}N^{2}-908m^{3}N+40mN+1057m^{4}-115m^{2}}{36{\left(N-3m\right)}^{2}{\left(m\tau_{0}\right)}^{4}}$$

for α=0,

$$\begin{array}{cc} \mathrm{Var}\left[{\hat{\sigma }}_{z}^{2}\left(m\tau_{0}\right)\right]= & \frac{q_{2}^{2}}{864{\left(N-3m\right)}^{2}m^{4}}\left(\frac{25}{84}N^{8}-\frac{80}{7}mN^{7}+\frac{956}{5}m^{2}N^{6}-15N^{6}-\frac{9098}{5}m^{3}N^{5}\right.\\ & +72mN^{5}+10770m^{4}N^{4}-860m^{2}N^{4}+\frac{25}{4}N^{4}-40584m^{5}N^{3}+5450m^{3}N^{3}\\ & -120mN^{3}+95052m^{6}N^{2}-19314m^{4}N^{2}+\frac{4304}{5}m^{2}N^{2}-\frac{85}{21}N^{2}\\ & -\frac{632348}{5}m^{7}N+36284m^{5}N-\frac{13564}{5}m^{3}N+\frac{416}{7}mN+\frac{2560783}{35}m^{8}\\ & -28228m^{6}+\frac{15871}{5}m^{4}-\left.\frac{1116}{7}m^{2}\right) \end{array}$$

for α=−2, and:

$$\begin{array}{cc} \mathrm{Var}\left[{\hat{\sigma }}_{z}^{2}\left(m\tau_{0}\right)\right]= & \frac{\tau_{0}^{4}q_{3}^{2}}{138240{\left(N-3m\right)}^{2}m^{4}}\left(\frac{5}{198}N^{12}-\frac{16}{11}mN^{11}\right.+\frac{344}{9}m^{2}N^{10}-\frac{35}{54}N^{10}-\frac{5450}{9}m^{3}N^{9}\\ & +\frac{280}{9}mN^{9}+\frac{45065}{7}m^{4}N^{8}-\frac{14045}{21}m^{2}N^{8}+\frac{755}{126}N^{8}-\frac{1015316}{21}m^{5}N^{7}+\frac{59320}{7}m^{3}N^{7}\\ & -\frac{4832}{21}mN^{7}+\frac{1313888}{5}m^{6}N^{6}-70034m^{4}N^{6}+\frac{57706}{15}m^{2}N^{6}-\frac{145}{6}N^{6}\\ & -\frac{5202144}{5}m^{7}N^{5}+\frac{1182328}{3}m^{5}N^{5}-\frac{548318}{15}m^{3}N^{5}+696mN^{5}+1487529m^{8}N^{4}\\ & -763918m^{6}N^{4}+215737m^{4}N^{4}-\frac{74801}{9}m^{2}N^{4}+\frac{1120}{27}N^{4}-5984488m^{9}N^{3}\\ & +4024112m^{7}N^{3}-\frac{2427188}{3}m^{5}N^{3}+\frac{473732}{21}m^{3}N^{3}-\frac{7168}{9}mN^{3}+\frac{40142892}{5}m^{10}N^{2}\\ & -6881802m^{8}N^{2}+\frac{9403928}{5}m^{6}N^{2}-\frac{1304218}{7}m^{4}N^{2}+\frac{39964}{7}m^{2}N^{2}-\frac{5240}{231}N^{2}\\ & -\frac{289902724}{45}m^{11}N+\frac{433989944}{63}m^{9}N-\frac{37117292}{15}m^{7}N+\frac{22002368}{63}m^{5}N\\ & -\frac{162080}{9}m^{3}N+\frac{23168}{77}mN+\frac{1618838030}{693}m^{12}-\frac{192734554}{63}m^{10}\\ & +\frac{29575226}{21}m^{8}-\frac{17042854}{63}m^{6}+\left.\frac{1331272}{63}m^{4}-\frac{60128}{77}m^{2}\right) \end{array}$$

for α=−4.

When 1/6≤m/N<1/5,

$$\mathrm{Var}\left[{\hat{\sigma }}_{z}^{2}\left(m\tau_{0}\right)\right]=\frac{\left(461N-1680m\right)q_{0}^{2}}{9{\left(N-3m\right)}^{2}{\left(m\tau_{0}\right)}^{4}}$$

for α=2,

$$\mathrm{Var}\left[{\hat{\sigma }}_{z}^{2}\left(m\tau_{0}\right)\right]=q_{1}^{2}\frac{\frac{1}{3}N^{4}-8mN^{3}+72m^{2}N^{2}-\frac{1}{3}N^{2}-232m^{3}N+88mN+228m^{4}-300m^{2}}{144{\left(N-3m\right)}^{2}{\left(m\tau_{0}\right)}^{4}}$$

for α=0,

$$\begin{array}{cc} \mathrm{Var}\left[{\hat{\sigma }}_{z}^{2}\left(m\tau_{0}\right)\right]= & \frac{q_{2}^{2}}{864{\left(N-3m\right)}^{2}m^{4}}\left(\frac{1}{84}N^{8}-\frac{4}{7}mN^{7}+12m^{2}N^{6}-\frac{1}{10}N^{6}-144m^{3}N^{5}\right.\\ & +\frac{18}{5}mN^{5}+1080m^{4}N^{4}-54m^{2}N^{4}+\frac{1}{4}N^{4}-5184m^{5}N^{3}+432m^{3}N^{3}\\ & -6mN^{3}+15552m^{6}N^{2}-1944m^{4}N^{2}+54m^{2}N^{2}-\frac{17}{105}N^{2}\\ & -\frac{932686}{35}m^{7}N+4684m^{5}N-\frac{982}{5}m^{3}N+\frac{824}{35}mN\\ & +\frac{698283}{35}m^{8}-4728m^{6}+\frac{1311}{5}m^{4}-\left.\frac{540}{7}m^{2}\right) \end{array}$$

for α=−2, and:

$$\begin{array}{cc} \mathrm{Var}\left[{\hat{\sigma }}_{z}^{2}\left(m\tau_{0}\right)\right]= & \frac{\tau_{0}^{4}q_{3}^{2}}{4147200{\left(N-3m\right)}^{2}m^{4}}\left(\frac{1}{33}N^{12}-\frac{24}{11}mN^{11}+72m^{2}N^{10}-\frac{7}{9}N^{10}-1440m^{3}N^{9}\right.\\ & +\frac{140}{3}mN^{9}+19440m^{4}N^{8}-1260m^{2}N^{8}+\frac{151}{21}N^{8}-186624m^{5}N^{7}+20160m^{3}N^{7}\\ & -\frac{2416}{7}mN^{7}+1306368m^{6}N^{6}-211680m^{4}N^{6}+7248m^{2}N^{6}-29N^{6}\\ & -6718464m^{7}N^{5}+1524096m^{5}N^{5}-86976m^{3}N^{5}+1044mN^{5}+25194240m^{8}N^{4}\\ & -7620480m^{6}N^{4}+652320m^{4}N^{4}-15660m^{2}N^{4}+\frac{448}{9}N^{4}-67184640m^{9}N^{3}\\ & +26127360m^{7}N^{3}-3131136m^{5}N^{3}+125280m^{3}N^{3}-\frac{3584}{3}mN^{3}\\ & +120932352m^{10}N^{2}-58786560m^{8}N^{2}+9393408m^{6}N^{2}-563760m^{4}N^{2}\\ & +10752m^{2}N^{2}-\frac{2096}{77}N^{2}-\frac{30472331996}{231}m^{11}N+\frac{235162420}{3}m^{9}N\\ & -\frac{112693988}{7}m^{7}N+\frac{28477444}{21}m^{5}N-\frac{121360}{3}m^{3}N+\frac{226752}{77}mN\\ & +\frac{5076178850}{77}m^{12}-\frac{329308930}{7}m^{10}+\frac{84470010}{7}m^{8}-\frac{9520870}{7}m^{6}\\ & +\frac{400840}{7}m^{4}-\left.\frac{720000}{77}m^{2}\right). \end{array}$$

for α=−4.
